# Application of noninvasive sampling technique in mitochondrial genome intraspecific phylogeny of the endangered butterfly, *Teinopalpus aureus* (Lepidoptera: Papilionidae)

**DOI:** 10.1093/jisesa/ieae008

**Published:** 2024-02-27

**Authors:** Wen-Jing Yang, Gui-Qiang He, Chao-Bin Huang, Shan-Yi Zhou, Feng-Hai Jia, Ju-Ping Zeng

**Affiliations:** Key Laboratory of National Forestry and Grass and Administration on Forest Ecosystem Protection and Restoration of Poyang Lake Watershed, College of Forestry, Jiangxi Agricultural University, Nanchang 330045, PR China; Jiulianshan Forest Ecosystem Observation Station, Longnan 341701, PR China; Jinggangshan National Nature Reserve of Jiangxi, Jinggangshan 343600, PR China; Nanning Institute of Termite Control, Nanning 530000, PR China; Key Laboratory of Ecology of Rare and Endangered Species and Environmental Protection, Ministry of Education, College of Life Science, Guangxi Normal University, Guilin 541006, PR China; Jiangxi University of Chinese Medicine, Nanchang 330004, PR China; Key Laboratory of National Forestry and Grass and Administration on Forest Ecosystem Protection and Restoration of Poyang Lake Watershed, College of Forestry, Jiangxi Agricultural University, Nanchang 330045, PR China; Jiulianshan Forest Ecosystem Observation Station, Longnan 341701, PR China

**Keywords:** *Teinopalpus aureus*, noninvasive sampling, DNA extraction, mitochondrial genome, intraspecific phylogeny

## Abstract

The butterfly genus of *Teinopalpus*, endemic to Asia, embodies a distinct species of mountain-dwelling butterflies with specific habitat requirements. These species are rare in the wild and hold high conservation and research value. Similar to other protected species, the genetic analysis of the rare *Teinopalpus aureus* poses challenges due to the complexity of sampling. In this study, we successfully extracted DNA and amplified mitochondrial genomic DNA from various noninvasive sources such as larval feces, larval exuviae, larval head capsules, pupal exuviaes, and filamentous gland secretions, all integral parts of butterfly metamorphosis. This was conducted as part of a research initiative focused on the artificial conservation of *T. aureus* population in Jinggang Shan Nature Reserve. Our findings illustrated the successful extraction of DNA from multiple noninvasive sources, achieved through modified DNA extraction methodologies. Although the DNA concentration obtained from noninvasive samples was lower than that from muscle tissues of newly dead larvae during rearing, all samples met the requirements for PCR amplification and sequencing, yielding complete circular sequences. These sequences are pivotal for both interspecific and intraspecific genetic relationship analysis. Our methods can be extended to other insects, especially scarce species.

## Introduction

Genetic diversity is the foundation of the Earth’s life system, and knowledge of changes in genetic diversity can provide insight into biological evolution at the microscopic level (Wang et al. 2017, Schmidt and Garroway 2021), understand species adaptations and their evolutionary potential, and capture the viability of geographic populations (Frankham 2005). However, delving into the genetic diversity of endangered and threatened species, particularly animals, presents a challenge in sample collection, since destructive methods are usually not welcomed in most regions.

Animal DNA sampling, categorized by Taberlet (1999), commonly includes 3 approaches: (i) traditional destructive sampling (DS), (ii) nondestructive sampling (NDS), and (iii) noninvasive sampling (NIS) (Taberlet et al. 1999). The DS method is abandoned by most researchers as it requires killing certain animal individuals. The NDS method involves minimally invasive collection of localized tissue samples (e.g., blood, muscle, etc.), often through capture or the use of tools like biopsy dart guns (Hoelzel and Amos 1988, Georgiadis et al. 1994, Pagano et al. 2014, Mijele et al. 2016). Conversely, the NIS method strives for nonintrusive, nondisturbance, noninvasive (to live animals or their specimens) sampling conditions. It exclusively relies on residual remnants from an animal’s life-cycle, such as excreted feces, shed feathers, or similar sources (Faria et al. 2011). This method opens a new pathway for genetic diversity research (Beja-Pereira et al. 2009, Carroll et al. 2018, Baus et al. 2019, Fuwen et al. 2021). However, it is critical to note that even when feathers are directly acquired from live animals or their specimens, it introduces a level of disruption, hence should be classified under NDS, not NIS (Taberlet et al. 1999). Likewise, in the context of insects, direct sampling from individuals, such as taking a foot from a dried specimen or extracting DNA from a small number of butterfly wing scale cuts (Lushai et al. 2000), strictly aligns with the NDS protocol.

Unlike mammals and birds, insects offer additional DNA sources for NIS beyond excreted feces, larval exuviae, and body shells (e.g., egg chorion, larval head capsule, pupal exuviaes, etc.) left behind during the metamorphosis serve as potential genetic resources. For examples, the high-quality DNA has been successfully extracted from the egg chorion of Miami blue butterfly, *Cyclargus thomas bethunebakeri* W.P Comstock & Huntington (Lycaenidae: Cyclargus) larvae, and from the larval exuviae (similar to feces) of *Vanessa cardui* (Linnaeus, 1758) (Nymphalidae: Vanessa) larvae, which, using these source, the ND1 gene sequence is amplified (Feinstein 2004, Storer et al. 2019). However, these sources have been neglected in DNA extraction and genetic diversity researches, especially in the field of conservation genetics for endangered insects, and the urgency remains for the development and application of more available NIS methods across a broader spectrum of insect taxa (Faria et al. 2011). Here, this study focuses on *Teinopalpus aureus* as a case study to explore some existing NIS methods applicable in endangered butterflies.

*Teinopalpus aureus*, known as the golden kaiserihind, is a large butterfly endemic to the tropical and subtropical regions of Asia (Zhou 1994, Igarashi 2001, Wu and Xu 2017, Zou et al. 2021b). Regarded as one of “Three Unusual Species of Swallowtail Butterflies” (Igarashi 2001), it has been listed as Red Species of IUCN since 1985 (Gimenez Dixon 1996) and designated as a first-class of National Key Protected Animals in China since 1989. Obviously, the nature of this species renders the application of DS or NDS methods unfeasible; hence, the NIS approach is highly anticipated by entomologists, conservation biologists, and amateurs. Their focus remains steadfast on the genetic diversity of 2 sister species in *Teinopalpus*, *T. aureus* and *T. imperialis* (Zhou 1994, Wu and Xu 2017), and their controversial supra- or infra-specific phylogenetics (Qin et al. 2011; Huang et al. 2015, 2016, Wang et al. 2018, Zou et al. 2021a, Liu et al. 2022). At present, the population occurrences of these 2 species are significantly constrained by the high-quality forests in mountains regions (Wang et al. 2022), especially amidst threats of global warming, aridification, and human exploitation (Zeng et al. 2005, 2012, Xing et al. 2019). For the conservation of *Teinopalpus*, it is a critical need to comprehend genetic diversity shifts, adaptability, evolutionary potential, and the viability of local populations, etc. Consequently, this case study on NIS endeavors to surmount the challenge of DNA sampling in endangered butterflies. It will facilitate the research facets within conservation genetics and molecular ecology (Beja-Pereira et al. 2009).

Given the mitochondrial genome’s characteristics such as (i) strict maternal inheritance (Gyllensten et al. 1985); (ii) relatively straightforward structure (Bibb et al. 1981); (iii) optimal size and evolutionary rate, as well as different conservativeness in each gene, etc. (Vawter and Brown 1986); and (iv) it is widespread application as a molecular marker in insect phylogenetic studies, particularly within Lepidoptera (Gray 1989, Niu et al. 2001, Daizhen et al. 2008, Dai et al. 2016). We attempted to extract DNA from different NIS samples of *T. aureus* in this study and analyzed the effects of source, preservation, and preserved duration on the DNA quality. Afterwards, we further conducted mitochondrial genome amplification, and by using these molecular markers, we performed intraspecific phylogenetic analysis on the unknown Jinggang Shan (JGS) (a mountain name) population of *T. aureus* in southern China, attempting to confirm the validity of NIS methods and provide a referable case for rare insects.

## Materials and Methods

### Samples and Preservation

The recently dead larvae of *T. aureus* originated from JGS National Nature Reserve (114°04ʹ05″–114°16ʹ38″E, 26°38ʹ39″–26°40ʹ03″N), where a butterfly artificial breeding experiment was conducted during 2019–2022. Various remnants such as larval exuviae, larval head capsules, feces, and silk gland secretion on the larval roosting leaves, etc. were collected as NIS sources. Additionally, postemergence, the chrysalis shells of the pupae were also utilized as NIS sources (Table 1).

**Table 1.** Noninvasive sample sources of DNA and preserved duration, methods in *T. aureus*

Sampling	Sources (abbreviations)	Preserved methods (abbreviations)	Preserved time^a^	Repetitions
Noninvasive	Larval head capsule (LvHC)	Silica gel dried-preserved (SGDP)	MT	3
	Larval exuviae (LvE)	Ethanol-preserved (EtP)	LT	3
		Dried-preserved (DrP)	LT	3
	Pupal exuviae (PpE)	Ethanol-preserved (EtP)	LT	3
		Silica gel dried-preserved (SGDP)	LT	3
	Larval silk gland secretion (LvSS)	Freeze-preserved (FrP)	MT	3
	Larval feces (LvF)	Silica gel dried-preserved (SGDP)	LT	3
		Silica gel dried-preserved (SGDP)	MT	3
		Dried-preserved (DrP)	MT	3
		Ethanol-preserved (EtP)	MT	3
		Freeze-preserved (FrP)	MT	3
		Fresh samples (Fresh)	ST	3
Invasive	Larval head (LvH)	Fresh samples (Fresh)	ST	3

^a^ST, short-term preserved (1–48 h); MT, middle-term preserved (within 1 month); LT, long-term preserved (≧18 months).

In DNA extraction experiments, we used the following samples preserved under different conditions: (i) the entire head of newly dead larvae; (ii) the fresh feces of the fourth or fifth instar larvae; (iii) the naturally dried larval exuviae and feces of the fourth or fifth instar; (iv) the larval exuviae and feces of the fourth or fifth instar as well as the pupal exuviaes preserved in 100% ethanol; (v) the larval head capsuls, feces of the fourth or fifth instar, the pupal exuviaes, and the secretion from the silk gland on leaves where larvae roosted, all preserved with the silica gel dried; and (vi) the frozen feces from fourth or fifth instar, stored at −20 °C. In DNA extraction, fresh samples were directly used, within a short period, specifically within 48 h, while other samples underwent middle-term, within 1 month, or long-term preservation for over 18 months. Detailed preservation timelines are outlined in Table 1.

### DNA Extraction and Detection

We used the Hipure Stool DNA Extraction Kit from Magen for the DNA extraction of larval feces. Approximately < 0.2 g fecal samples were placed in a 2-ml EP tube, mix with buffer ATL, and ground for 10 min in a bead mill. Subsequently, we followed the operation steps indicated by the Kit’s instruction. For other samples, we also used the DNeasy Blood and Tissue Kit from QIAGEN. While adhering strictly to the provided instructions, we optimized in certain steps to enhance the DNA yield from these noninvasive samples. For instance, we prolonged the initial incubation period up to 4 h at 55 °C, after the addition of lysis buffer and proteinase K. Following this, the incubated samples were then stored in a −20 °C refrigerator for at least 12 h before being returned to the 55 °C water bath, where the second incubation period lasted up to 2 h. Upon completion of all steps listed in the instruction manual, we quantified the DNA concentration and purity of each sample using an ultra-micro UV spectrophotometer (Thermo).

### PCR Amplification and Detection, Purification, DNA Sequence

We adhered to the methodology detailed by Huang (2016) for the PCR amplification of the mitochondrial gene in *T. aureus*, utilizing 21 primer pairs (see Table 2). Each 50-μl reaction system consisted of 2 μl of template DNA, 25 μl of 2× Hieff PCR Master Mix, 2 μl of upstream primers and downstream primers (at a concentration of 10 μmol/liter), and supplemented with dd H_2_O to reach the final volume of 50 μl.

**Table 2.** The 21 primer pairs’ names and sequence in *T. aureus*

Primers	Sequence (5ʹ–3ʹ)	Target fragment length/bp	Reaction procedure	Annealing temperature (°C)
TA1	CTTTTGGGCTCATACCTC TATCATAATAAAATTATGGGG	673	1	49.3
TA2	ATCTTTTATCATCAGAAGCAGC GTTCCAATATCTTTATGGTTTG	1,099	1	52.8
TA3	TTCGAATTATTTATTCCTCT GTAATAAAATTAATGGCACCT	835	1	41.0
TA4	GGTCAACTCATAAAGATATTGG TAAACTTCAGGGTGACCAAAAAATCA	724	1	48.0
TA5	AGGTGGAGCCATTACGATAC AATAGCTGGGATAACGGTTC	1,215	1	54.5
TA6	TTTAGAAATGGCAACTTGA AAAATAAATTGTCGGTTAGGAA	1,115	1	51.0
TA7	TATTCCTCAAATAATACCAA AGGAGGTCATATAATTCCA	1,187	1	51.0
TA8	ACGAGATATTTGCCGAGA AAATTAGGGCAATTTCAACA	901	2	43.0
TA9	GGCAGCTTGATATTGACATT TACCAAATTTATCGACTTATGGA	1,129	2	43.0
TA10	TATCTTCAATATCATGCTCT ATTGACAATGTTTATGGCTG	1,013	2	43.0
TA11	AATTCCACATAAAGACAAA TTTTATACAGGTATTTCTCG	1,233	2	44.0
TA12	AAAAGAAATAATTTCCCACTC ATTATTTGCTTCTTTACCGAT	1,099	1	49.3
TA13	TTAGGTAATCATAAATGAACAA ATATTCCTGATAAAAGGCAAG	1,172	2	42.0
TA14	TCTCTCTATCAATAATCTCC GGTAATTACTGTAGCACCT	1,039	2	38.0
TA15	ATTCACATATTGGACGAGGA TTTATTTGAGTTACGGGGAC	1,065	1	45.2
TA16	TGAGCGTGTTCAAGCGTTTG TGAGCCAGGTGAGTTTCCATCT	1,194	1	46.0
TA17	AACACCAATTAATACCCCTAA AAATAATATAAAGTCTAATCTGC	777	1	45.0
TA18	AATTACGCTGTTATCCCTAAG TAAAGAGAAAATATTTAATGGGG	1,052	1	50.0
TA19	ATTTATTTTAAAGCTTATCCT ACAATTGATAATCCACGAA	838	1	40.0
TA20	TTCAATTTATATATGAAAGCG TTTACATATAAATTTTAGTGTT	691	1	47.4
TA21	TTAAATTATTATTTGTATAACCG TTATATTTTAGTGTAAGATGC	800	1	52.0

Two separate reactions were conducted using the Heal Force T960 PCR instrument. The first reaction involved initial denaturation at 94 °C for 5 min, followed by 35 cycles at 94 °C for 45 s, 35–54.5 °C for 45 s, 70 °C for 90 s, and a final extension step of 72 °C for 10 min. The PCR products were stored at 4 °C. The second reaction also commenced with initial denaturation at 94 °C for 5 min, followed by 15 cycles at 94 °C for 50 s, 35–54.5 °C for 50 s, and 65 °C for 3 min, it involved 15 cycles at 94 °C for 55 s, 35–54.5 °C for 55 s, 68 °C for 185 s, and a final extension step at 68 °C for 10 min. The products from this round were also stored at 4 °C.

The PCR products were examined by electrophoresis on a 1% agarose gel and visualized photographed with the CLiNX gel imaging system (Shanghai Qin Xiang Scientific Instruments Co., Shanghai, China). Following the identification of distinct and well-defined bands, these samples were sent to the Qingdao Biotechnology Changsha Branch for gelatinization and purification. Subsequently, direct sequencing was conducted employing both forward and reverse primers on the ABI 3730 gene analyzer.

The complete mitochondrial genome of 3 mitochondrial genes from the *T. aureus* JGS population have been recorded in the GenBank/EMBL/DDBJ database with the GenBank accession number OR125018 (*T. aureus* JGS-3); OR251123 (*T. aureus* JGS-4); and OR253327 (*T. aureus* JGS-6).

### Sequence Splicing, Annotation, and Phylogenetic Analysis

MEGA7.0 software was used to verify the target gene sequence in the raw sequence files. The Seq Man software in DNA STAR was used in proofreading and assembly to obtain the complete mitochondrial genomic DNA. Also, from NCBI (https://www.ncbi.nlm.nih.gov), we downloaded all published *Teinopalpus* mitochondrial gene sequences (Qin et al. 2011, Huang 2016), and with the aid of MITOS online website (MITOS Web Server [uni-leipzig.de]), we completed mitochondrial genome annotations (Li et al. 2013).

The sequence base composition was analyzed by MEGA7.0 software, and Excel was employed to calculate the offset coefficients of AT and GC (AT-skew = (A − T)/(A + T), GC-skew = (G − C)/(G + C)). Using mitochondrial genomic data from different geographical populations published by NCBI, maximum likelihood (ML) and Bayesian (BI) phylogenetic trees were constructed. *Teinopalpus imperialis* and *Meandrusa sciron* were used as outgroups in this analysis.

For ML tree construction, the IQ-TERR software (Nguyen et al. 2017) was utilized. Parameters were set to MFP with the optimal model automatically set to TIM2 + F + G4, and 1,000 repetitions were performed to generate the phylogenetic tree.

The BI method was done in MrMtgui and MrBayes (Ronquist and Huelsenbeck 2003). Modeltest, PAUP, and Mrmodeltest were integrated with the model calculation software in MrMtgui. The optimal Akaike information criterion (AIC) standard model was calculated to be GTR + G after parameter adjustment based on the optimal model. MrBayes software was run with 4 Markov chains (MCMC) over 10 million generations, saving data every 1,000 generations. The first 25% of the tree was discarded, and the run was stopped when *P* ≤ 0.001 to obtain the BI phylogenetic tree. Tracer software was utilized to check the effective sample size (ESS) values; a value above 200 indicates ideal parameter convergence, ensuring the reliability of the evolutionary tree file. If needed, adjustments to parameters or an increase in running generations were made until optimal parameter convergence was achieved.

Finally, Fig Tree software was used to visualize and organize the results of different tree-building methods, offering tools for enhancing and editing phylogenetic trees.

### Data Analysis

The multivariate variance analysis was performed to investigate the effects among the sample sources, preservation, and preserved duration on DNA extraction concentration and quality. One-way ANOVA was used to detect the impact of preservation methods or time on DNA concentration in the larval feces. To compare independent samples from different sources or under different preservation methods, *t*-test was conducted. Prior to analysis, the data were ARTAN transformed. All statistical analyses were carried out using SPSS Statistics 17.0.

## Results and Analysis

### Concentration and Purity of DNA Extracted From Noninvasive Samples

#### Effects of sample sources, preservation and preserved duration on the concentration and quality of DNA.

**Fig. 1.**
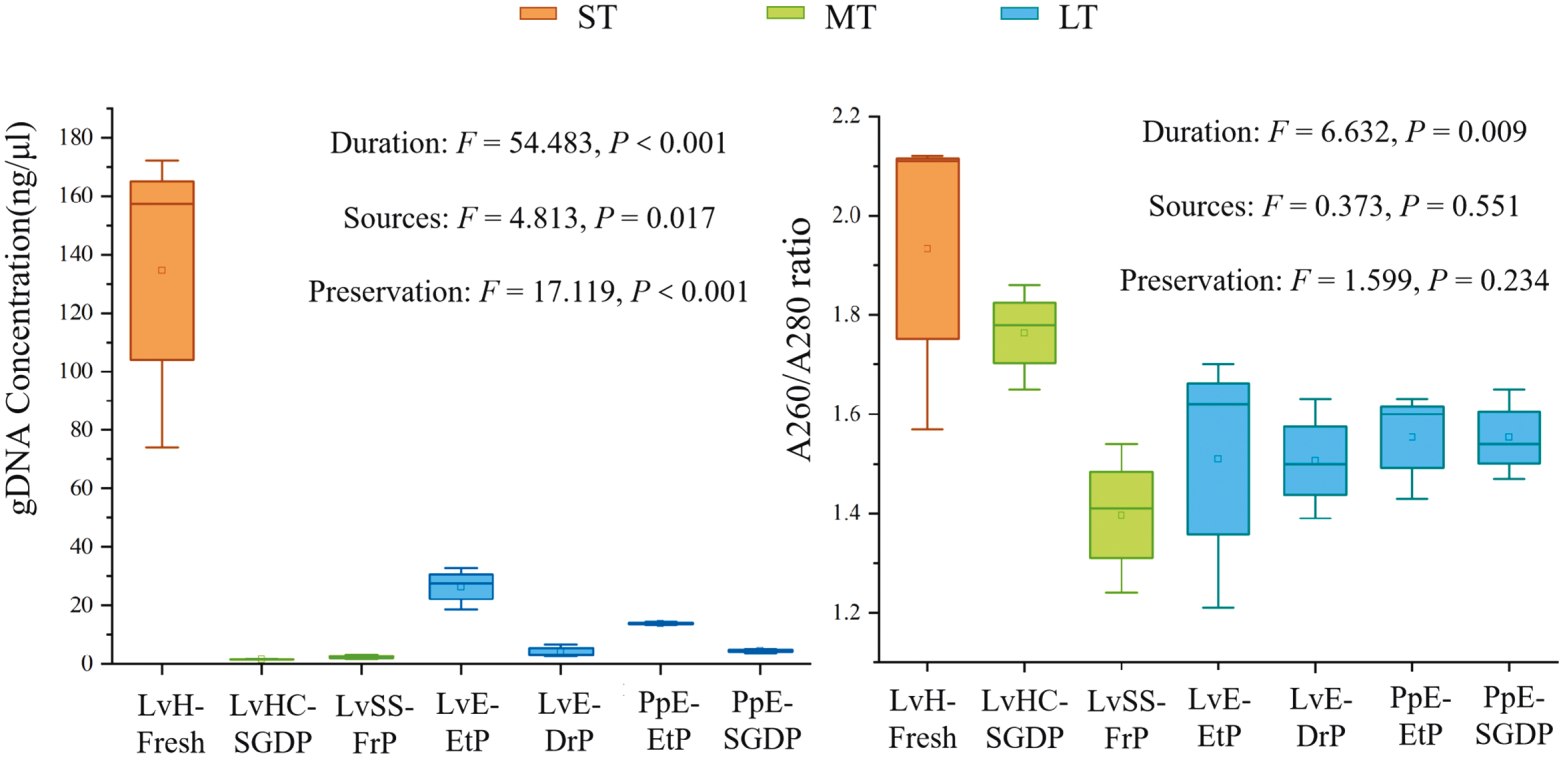
Comparison of genomic DNA concentration and purity among invasive (LvH: larval head) and noninvasive (LvE: larval exuviae; PpE: pupal exuviae; LvSS: larval silk gland secretions; LvHC: larval head capsule) samples of the butterfly *T. aureus*, under different preservations (SGDP: silica gel dried-preserved; EtP: ethanol-preserved; FrP: freeze-preserved; DrP: dried-preserved) and preserved duration, including ST, short-term preserved (1–48 h); MT, middle-term preserved (within 1 month), and LT, long-term preserved (≧18 months).

Figure 1 shows that the genomic DNA was successfully extracted from most noninvasive samples of the butterfly *T. aureus*. And results in Tables 3 and 4 indicated significant effects of sample sources (*P* = 0.017), preservation (*P* < 0.001), and preserved duration (*P* < 0.001) on genomic DNA concentration, while only the preserved duration (*P* = 0.006) significantly affected the DNA purity. The invasive sample of larval head provided the highest concentration of 134.5 ± 52.8 ng/µl (Fig. 1), and among different noninvasive samples, the DNA concentration was up to 26.4 ± 7.23 ng/µl from larval exuviae (Fig. 1).

**Table 3.** Effects of sample sources, preservation, and preserved duration on genomic DNA concentration in multivariate variance analysis^a^

Sources	Type I sum of squares	df	Mean square	*F*	*P*
Corrected model	42,014.585^b^	6	7,002.431	17.193	<0.001
Intercept	15,064.286	1	15,064.286	36.987	<0.001
Preserved duration	22,189.781	1	22,189.781	54.483	<0.001
Sources	5,880.262	3	1,960.087	4.813	0.017
Preservation	13,944.542	2	6,972.271	17.119	<0.001
Error	5,701.952	14	407.282		
Total	62,780.822	21			
Corrected total	47,716.537	20			

^a^With sources and preservation as fixed variables and preserved time as a covariate.

^b^ *R* ^2^ =0.881 and adjust *R*^2^ =0. 829.

**Table 4.** Effects of sample sources, preserved methods, and preserved time on genomic DNA purity in multivariate variance analysis^a^

Sources	Type I sum of squares	df	Mean square	*F*	*P*
Corrected model	0.692^b^	6	0.115	3.073	0.039
Intercept	52.583	1	52.583	1,400.067	<0.001
Preserved duration	0.393	1	0.393	10.452	0.006
Sources	0.206	3	0.069	1.826	0.189
Preservation	0.094	2	0.047	1.252	0.316
Error	0.526	14	0.038		
Total	53.801	21			
Corrected total	1.218	20			

^a^With sources and preservation as fixed variables and preserved time as a covariate.

^b^ *R* ^2^ =0.568 and Adjust *R*^2^ =0.383.

**Fig. 2.**
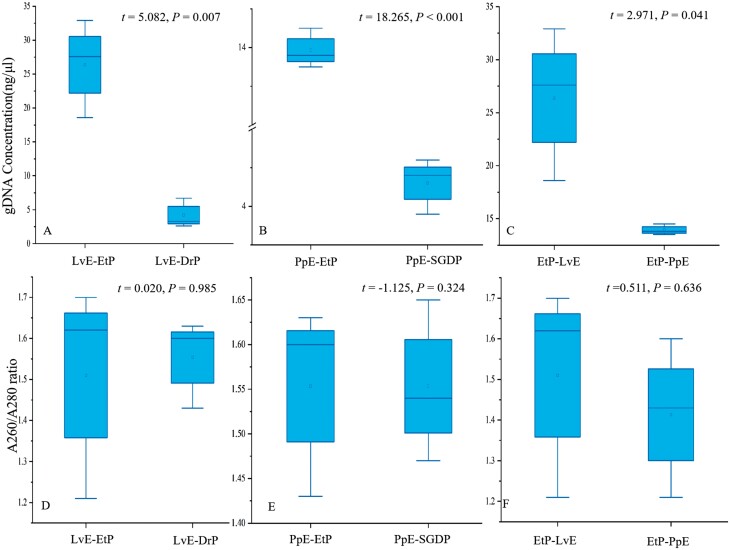
Comparison of genomic DNA concentration and purity between the long-term preserved (≧18 months) noninvasive samples of larval exuviae (LvE) and pupal exuviae (PpE) of the butterfly *T. aureus*, under different preservations (SGDP: silica gel dried-preserved; EtP: ethanol-preserved).

As for noninvasive samples, Fig. 2 indicated that the ethanol-preserved samples always provided the better DNA concentration, either in the case of larval exuviae (Fig. 2A, *t* = 5.082, *P* = 0.007) or pupal exuviae (Fig. 2B, *t* = 18.265, *P* < 0.001). For example, the DNA concentration was 26.37 ± 7.23 ng/μl in alcohol-preserved larval exuviae, while it was low to 4.2 ± 2.12 ng/μl for the desiccation-preserved one. But to be noted, the concentration of DNA obtained from the larval exuviae was significantly higher than that from pupal exuviae (Fig. 2C, *t* = 2.971, *P* = 0.041). However, in these noninvasive samples, Fig. 2C–F showed no significant differences observed for the DNA purity, either among different preservation methods or among different sources.

#### Effects of preservation and preserved duration on the concentration and quality of genomic DNA extracted from larval feces.

**Fig. 3.**
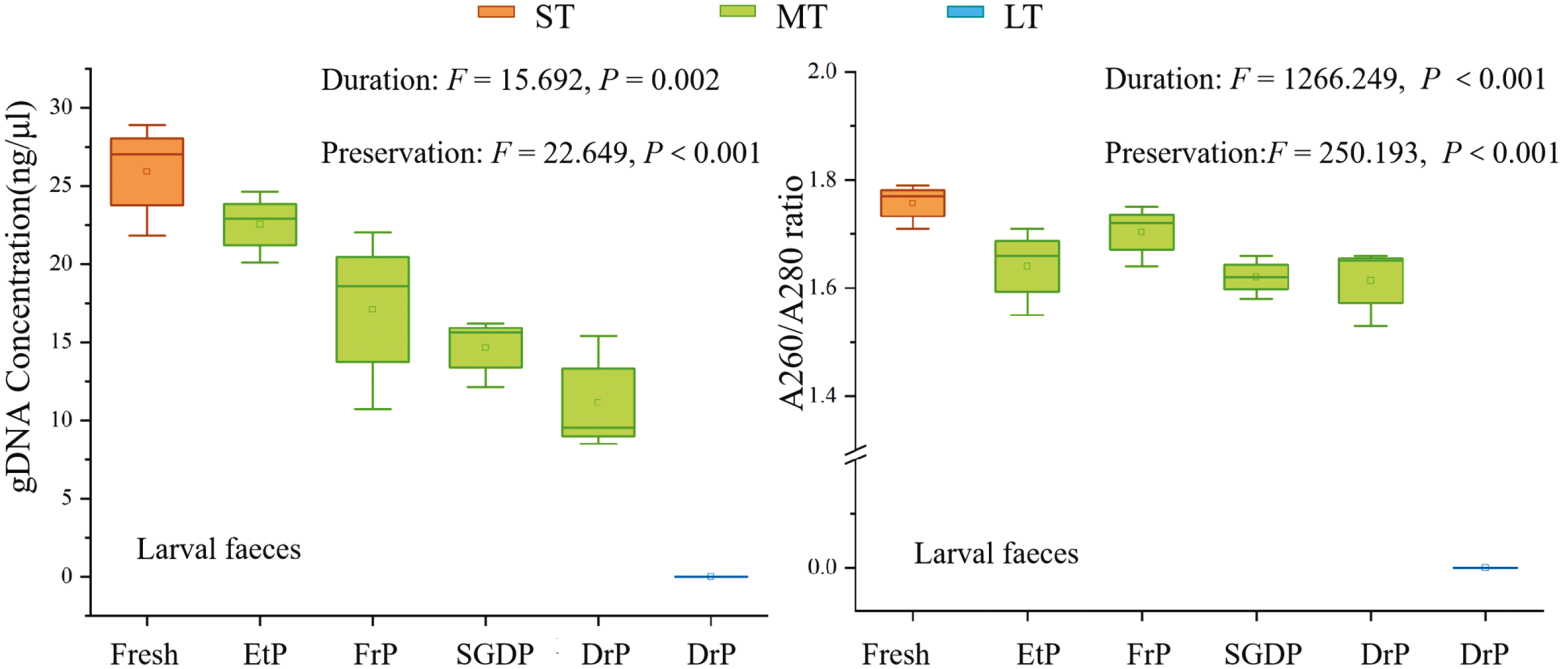
Comparison of genomic DNA concentration and purity of larves feces, under different preservations (EtP: ethanol-preserved; FrP: freeze-preserved; SGDP: silica gel dried-preserved; DrP: dried-preserved) and preserved duration, including ST, short-term preserved (1–48 h); MT, middle-term preserved (within 1 month) and LT, long-term preserved (≧18 months).

In Fig. 3, the genomic DNA was extracted from all larval feces samples, except for the naturally-dried sample stored in a long term, and it revealed significant effects of preservation (*F* = 22.649, *P* < 0.001) and preservation duration (*F* = 15.692, *P* = 0.002) on feces DNA concentration (Fig. 3). The fresh feces provided the highest concentration, and among other samples stored for a middle term, the ethanol-preserved feces performed better in DNA concentration, followed the freeze-preserved and silica gel dried-preserved feces. However, the purity of DNA extracted from feces varied little among samples stored with different preservations (Fig. 3), when excluded the long-term preserved sample.

### PCR Amplification Products and Mitochondrial Genome

#### PCR amplification products of noninvasive samples.

**Fig. 4.**
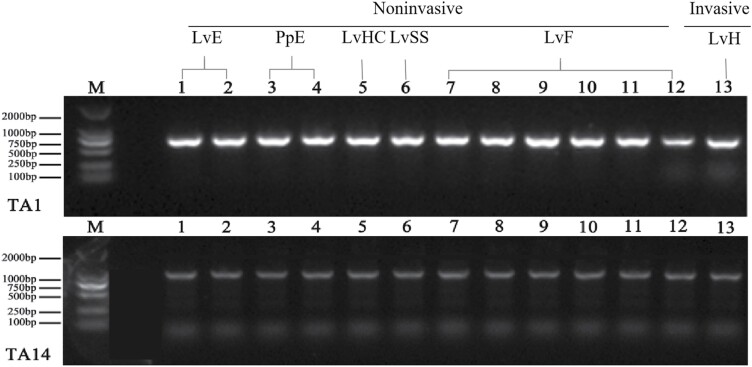
Electropherogram of PCR amplification products of the 2 mitochondrial DNA fragments (TA1 and TA14, see Table 2) in noninvasive (LvE: larval exuviae; PpE: pupal exuviae; LvHC: larval head capsule; LvSS: larval silk gland secretions; LvF: larval feces) and invasive samples (LvH: larval head), M: DL2000 DNA Marker.

The electrophoresis map in Fig. 4 inspected the PCR amplification products of 2 mitochondrial DNA fragments (TA1 and TA14, see Table 2) in *T. aureus*, and it showed well that the amplification results from the noninvasive samples were as stable as that from invasive samples, since all the objective bands were correct and clear (Fig. 4).

#### Mitochondrial genome structure and sequence length variation.

**Fig. 5.**
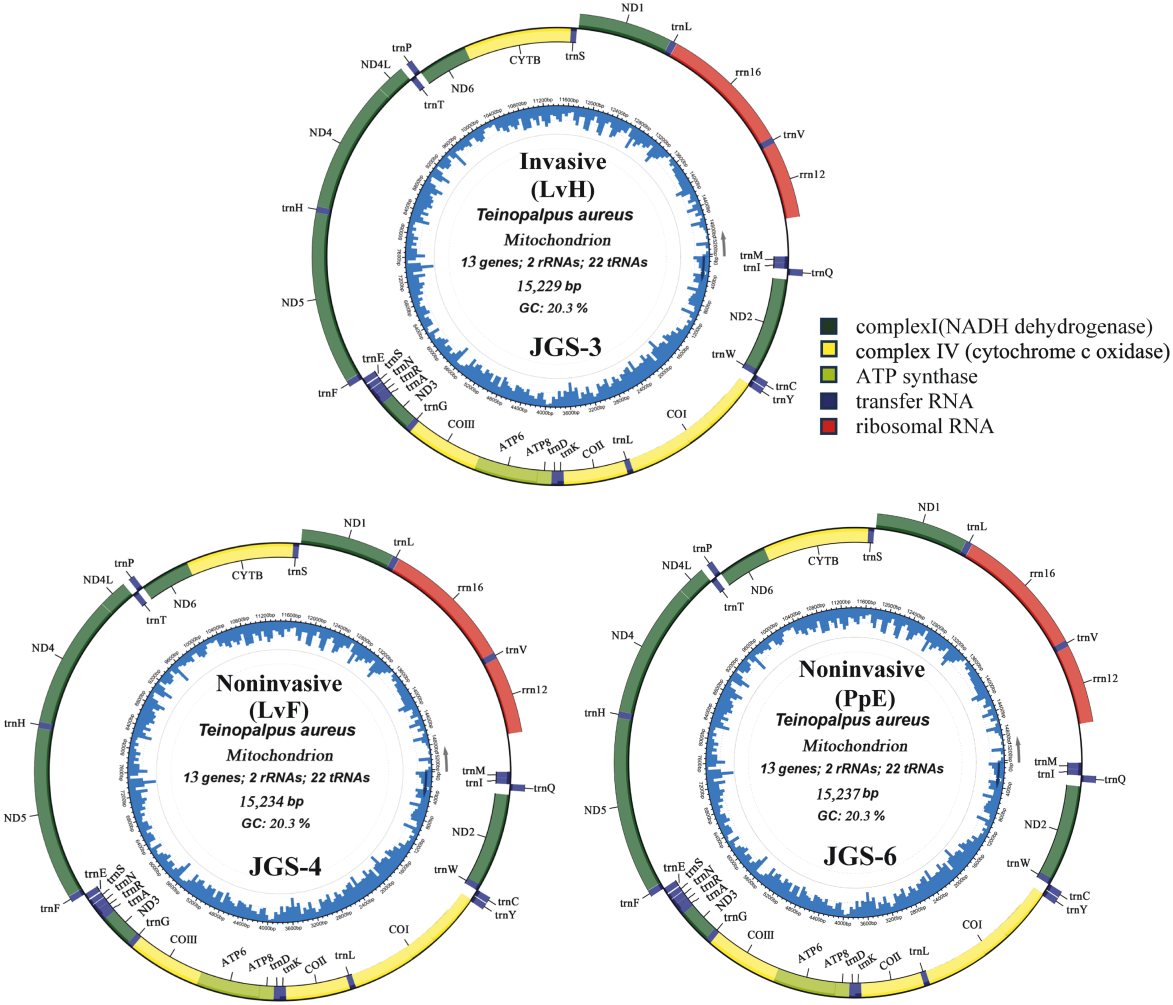
Three circular mitochondrial DNA from noninvasive (LvF: larval feces; PpE: pupal exuviae) and invasive (LvH: larval head) sample of *T. aureus* in Jinggangshan, South China.

**Fig. 6.**
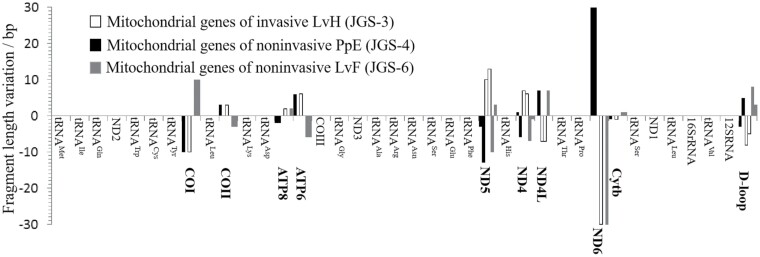
Fragment length variation of mitochondrial genes from noninvasive (LvF: larval feces; PpE: pupal exuviae) and invasive (LvH: larval head) samples of *T. aureus* in Jinggangshan, South China.

In Fig. 5, it showed the 3 circular mitochondrial DNA sequences of *T. aureus* in Jinggangshan, namely JGS-3, JGS-4, and JGS-6. The JGS-3 was obtained from an invasive sample of larval head (LvH), and the other 2 were obtained from 2 noninvasive samples, of larval feces (LvF) and pupal exuviae-(PpE). The 3 sequences all comprised 37 genes, including 13 protein-coding genes (COI-III, ND1-6, ATP6, ATP8, Cytb, ND4L), 2 rRNA genes (12s rRNA vs. 16s rRNA), 22 tRNA genes, and 1 noncoding control region (D-loop) (Table 5, Fig. 5). The sequence length varied slightly across these 3 mitochondrial DNAs, for instance, 15,237 bp in JGS-6, 15,234 bp in JGS-4, and 15,229 bp in JGS-3 (Fig. 5), and from the fragment length variation in Fig. 6, it implied that the identical 10 loci (including COI, COII, ATP8, ATP6, ND5, ND4, ND4L, ND6, Cytb, and D-loop) could mainly contribute such sequence length variation, since these fragment lengths always kept a consistent variation among the 3 mitochondrial DNAs.

**Table 5.** Mitochondrial genes structure and characteristics of *T. aureus* from larval feces (LvF) in Jinggangshan, South China

Genes	Direction	Nucleotide location	Fragment length/bp	Interval	Start codon	Termination codon
tRNAMet	F	1–70	70	0		
tRNA^Ile^	F	71–134	64	−3		
tRNA^Gln^	R	132–200	69	−9		
ND2	F	248–1,261	1,014	−42	ATT	TAA
tRNA^Trp^	F	1,260–1,324	65	−8		
tRNA^Cys^	R	1,317–1,382	66	8		
tRNA^Tyr^	R	1,391–1,457	67	5		
COI	F	1,463–2,989	1,526	−5	CGA	T-tRNA^Leu^
tRNA^Leu^	F	2,990–3,056	67	0		
COII	F	3,058–3,742	685	−3	ATG	tRNA^Lys^
tRNA^Lys^	F	3,739–3,809	71	−1		
tRNA^Asp^	F	3,809–3,875	67	0		
ATP8	F	3,876–4,033	157	−7	ATT	TAA
ATP6	F	4,027–4,704	678	−1	ATG	TAA
COIII	F	4,704–5,492	789	−1	ATG	TAA
tRNA^Gly^	F	5,492–5,557	66	0		
ND3	F	5,558–5,911	354	−2	ATT	TAG
tRNA^Ala^	F	5,910–5,975	66	−1		
tRNA^Arg^	F	5,975–6,038	64	0		
tRNA^Asn^	F	6,039–6,106	68	5		
tRNA^Ser^	F	6,112–6,171	60	13		
tRNA^Glu^	F	6,185–6,250	66	−2		
tRNA^Phe^	R	6,249–6,314	66	2		
ND5	R	6,317–8,052	1,735	5	ATA	TAA
tRNA^His^	R	8,057–8,121	65	−20		
ND4	R	8,122–9,460	1,339	2	ATG	TAA
ND4L	R	9,463–9,753	291	2	CTA	TTA
tRNA^Thr^	F	9,756–9,820	65	0		
tRNA^Pro^	R	9,821–9,885	65	32		
ND6	F	9,888–10,421	534	−1	ATT	TAA
Cytb	R	10,421–11,567	1,146	−2	ATG	TAA
tRNA^Ser^	F	11,566–11,630	65	16		
ND1	F	11,647–12,585	939	1	ATG	TAG
tRNA^Leu^	F	12,587–12,654	68	−25		
16SrRNA	R	12,630–13,962	1,333	30		
tRNA^Val^	R	13,993–14,058	66	0		
12SrRNA	R	14,059–14,824	766	10		
D-loop	R	14,825–15,234	409	0		

### Phylogenetic Analysis of Mitochondrial Genomes From Noninvasive Samples

#### Base composition and sequence variation.

We conducted a comparative analysis of the mitochondrial genomes of *T. aureus* from Jingang Shan with data obtained from 4 geographical populations available in NCBI (WYS-MW900433, MHS-KP941016, PS-KP941017.1, DYS-KP941013) using MEGA7.0. This analysis revealed 13,272 homologous sites, including 13,098 conservative sites (C), 105 variant sites (V), and 66 parsimony informative sites (P). The average base content of A, T, C, and G were 39.7%, 40.1%, 12.6%, and 7.7%, respectively (Table 6), indicating a notable bias toward A and T, constituting 79.8% of the A + T nucleotides. This bias was notably prevalent in the protein-coding genes, rRNA genes, and tRNA genes, especially prominent in the D-loop region, reaching 93.1%. Additionally, the AT-skew and GC-skew values highlighted significant differences in the usage of AT or GC, with the most pronounced variance observed in the control or D-loop region (AT-skew = −0.0655, CG-skew = −6.1765; in Table 6).

**Table 6.** Mitochondrial genome’s base composition and skewness of *T. aureus*, from noninvasive (larval feces-LvF, pupal exuviae-PpE) and invasive (larval head-LvH) samples in Jinggangshan, South China

Base and skewness	mtDNA	PCGs	rrnS	rrnL	tRNA gene	Control region
A	39.7	39.1	42	40.9	41.2	43.5
T	40.1	39.2	44.2	41.3	39.3	49.6
C	12.6	13.3	9.2	12.7	12.2	5.3
G	7.7	8.5	4.6	5.2	7.3	1.5
A + T	79.8	78.3	86.2	82.2	80.5	93.1
AT-skew	−0.0050	−0.0013	−0.0255	−0.0049	0.0236	−0.0655
GC-skew	−1.5764	−1.4037	−2.7101	−1.9944	−1.7385	−6.1765

#### Phylogenetic analysis.

**Fig. 7.**
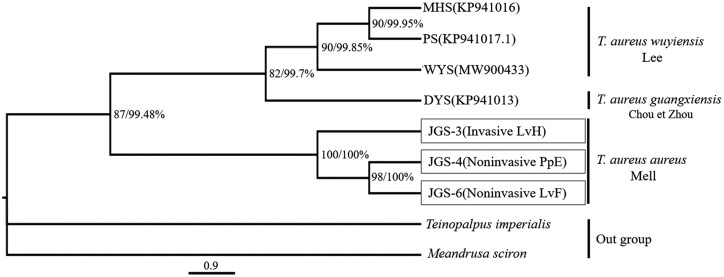
Maximum likelihood (ML) and Bayesian (BI) phylogenetic tree constructed with 13 protein-coding gene sequences of mitochondrial genes of *T. aureus*, by using *T. imperialis* and *Meandrusa sciron* as an outgroup, showing ML 1,000 cycles of bootstrap test and BI posterior probability values, as well as the invasive (LvH: larval head) and noninvasive (LvF: larval feces; PpE: pupal exuviae) samples from Jinggangshan, South China.

The ML and BI phylogenetic trees in Fig. 7 demonstrate that JGS-3, JGS-4, and JGS-6 formed a distinct cluster with high bootstrap values. This clustering remained consistent across invasive (larval head-LvH) or noninvasive (larval feces-LvF, pupal exuviae-PpE) samples. Notably, the trees clearly delineate the geographical population of Jinggang Shan, showing its affiliation with the nominate subspecies of *T. aureus aureus* Mell. This separation is evident from the clustering, which distinctly separates it from another cluster consisting of *T. aureus wuyiensis* Lee (e.g., MHS, PS, WYS) and *T. aureus guangxiensis* Chou et Zhou (e.g., DYS) (Fig. 7).

## Discussion

Insect feces, like birds’ and mammals’ excreta, contain exfoliated gut wall cells and serve as potential NIS samples for DNA extraction, despite their relatively small size. Apart from fecal DNA, which involves food residue mixed with excreta (Ali et al. 2019), larval exuviae and body shells discarded during insect metamorphosis are also valuable NIS samples, particularly in rare or endangered species. This study explored the possible NIS samples of the endangered butterfly *T. aureus*, including larval exuviae, pupal exuviae, larval silk gland secretion, and larval feces. Genomic DNA was successfully obtained from most of these samples, enabling stable amplification and unveiling the mitochondrial genome structure, shedding light on the phylogenetic affiliation of the Jinggang Shan population to the nominate subspecies of *T. aureus aureus.*

Insects leave various body shells during their metamorphosis and development, such as egg chorion, larval exuviae, pupal exuviae etc., which all hold potential for DNA extraction. For example, Storer et al. (2019) successfully extracted DNA of sufficient quantity and quality for the single-gene sequencing from the chorion of residual butterfly egg debris left behind by the newly hatched larvae of the Miami blue butterfly *Cyclargus thomasi bethunebakeri* (Feinstein 2004). They also obtained DNA from the frass and exuviae of *Vanessa virginianus* and *Vanessa cardui*, generating good-quality sequences that confirm the utility of caterpillar frass and exuviae as DNA sources. In this study, we successfully extracted DNA from both larval exuviae and pupal exuviae of the butterfly *T. aureus*; however, egg chorion was not utilized. Furthermore, Donald et al. (2012) indicate that hemolymph-rich defensive secretions provide high-quality DNA. They extracted hemolymph from the defensive secretion of *Bolitotherus cornutus* (Panzer) (Coleoptera: Tenebrionidae), amplifying microsatellites from a large number of individuals, without adverse effects on mortality, defense response, or reproduction. Similarly, we explored the potential of larval silk gland secretion left on the leaf surface as a NIS sample in the butterfly of *T. aureus*. We successfully extracted the demanded DNA concentration for PCR amplification. In contrast to the passive and potentially negative nature of defensive secretion, the silk secretion left actively by larvae throughout the feeding period was completely unrestricted for sampling. Therefore, applying silk secretion as a sample could be more practical and available for DNA extraction of silking insects, further promote the application of the NIS method in insect conservation genetics and molecular ecology.

The DS method directly uses tissues, muscle, blood, etc., to extract DNA, whereas the NIS method relies on shed tissues as a source of DNA. For example, fecal DNA mainly consists of exfoliated cells from the gut wall (Sidransky et al. 1992), defensive secretion DNA is derived from the hemolymph carried by the secretions (Pan et al. 2004), and shell DNA originates from a small number of cells or cellular outgrowths shed during the molting process (Xu et al. 2001). Consequently, the DNA concentration obtained through the NIS method is usually lower due to the limited number of cells. For example, compared to the DNA originated from the entire head of newly dead larvae (DS method), the concentration from all NIS samples was significantly lower, whether derived from larval feces or pupal exuviaes, larval exuviaes, or the silk gland secretions. Therefore, this study appropriately optimized the standard experimental procedure.

Efforts were made to enhance DNA by optimizing the standard procedure. For instance, prolonging the first incubation time up to 4 h (from 2 h) under a 55 °C water bath, following by placing all the incubation samples in a −20 °C refrigerator for at least 12 h (Storer et al. 2019), and returning them back to the 55 °C water bath again for a 2-h incubation, etc. These modifications effectively promoted cell lysis, consequently increasing the DNA concentration to meet the requirements of PCR amplification for mitochondrial DNA markers. Further improvements in the insect NIS method are anticipated, such as the utilization of flow cytometry or FACS. These technologies are beneficial for generating a high-coverage, minimally biased mammalian genome solely from fecal samples, in addition to a low-coverage SNP dataset suitable for population assignment and clustering (Orkin et al. 2021).

Similar to the DS method, the quality of DNA extraction through the NIS method is also influenced by the preservation method and the duration of storage time (Tan and Wang 2008). For example, DNA quality derived from NIS samples decreases rapidly with prolonged storage; different preservation methods for noninvasive samples significantly impact the quality of DNA extraction. In the case of larval feces, fresh feces yielded superior DNA quality compared to the samples preserved. Extended conservation periods are associated with reduced DNA quality across various preservation methods, including alcohol storage, silica gel drying, and frozen storage. Generally, DNA extracted from samples preserved in absolute alcohol exhibited significantly better quality than subjected to silica gel drying and frozen storage. The degree of DNA degradation in this study followed the order of air drying > silica gel drying > 100% ethanol > fresh, aligning closely with findings from previous studies (Cai et al. 2006).

Nevertheless, DNA extracted by NIS method meets the criteria for mitochondrial molecular markers akin to DNA extracted by the DS method. Consequently, it becomes feasible to analyze genetic material diversity and related research fields. For example, mitochondrial genes of the JGS population of *T. aureus* obtained through the NIS method exhibited remarkable congruence with those acquired using the DS method from the same geographic area. The arrangement and coding directions of the 37 genes were consistent with reported geographical populations (Huang et al. 2015, Huang 2016, Zou et al. 2021a). Notably, the nucleotide composition in this genome shows a pronounced bias toward A + T. Phylogenetic analysis reveals that JGS-3, JGS-4, and JGS-6 cluster together with high bootstrap values, indicating identical mitochondrial genome sequences among these 3 samples from noninvasive (LvH, PpE) and invasive sampling (LvH) sampling methods within the same geographic population. Therefore, the results obtained through nondestructive methods signify that the JGS population of *T. aureus* is assigned to the named subspecies *T. aureus aureus* Mell, distinguished itself from *T. aureus wuyiensis* Lee (MHS, PS, WYS) and *T. aureus guangxiensis* Chou et Zhou (DYS). The established nondestructive sampling method for *Teinopalpus*, is well-suited for molecular identification and mitochondrial genetic analysis, consistently providing reliable results. This methodology can be extended to other rare Lepidoptera species or other insects, effectively addressing challenges in integrating molecular identification with genetic diversity studies and broadening the tools for molecular ecology research on rare butterflies (Srivathsan et al. 2016).

## Data Availability

The datasets generated during and analyzed during the current study are available from the corresponding author upon reasonable request.
